# The Pathogenesis of Central and Complex Sleep Apnea

**DOI:** 10.1007/s11910-022-01199-2

**Published:** 2022-05-19

**Authors:** Erin Grattan Roberts, Janna R. Raphelson, Jeremy E. Orr, Jamie Nicole LaBuzetta, Atul Malhotra

**Affiliations:** 1 Department of Medicine, University of California San Diego, 9500 Gilman Drive, La Jolla, San Diego, CA 92037 USA; 2 Division of Critical Care, Sleep Medicine and Physiology, Department of Medicine, University of California San Diego, San Diego, CA USA; 3 Division of Neurocritical Care, Department of Neurosciences, University of California San Diego, San Diego, CA USA

**Keywords:** Lung, Apnea, Sleep, Hypoxia, Obstructive, Central

## Abstract

**Purpose:**

The purpose of this article is to review the recent literature on central apnea. Sleep disordered breathing (SDB) is characterized by apneas (cessation in breathing), and hypopneas (reductions in breathing), that occur during sleep. Central sleep apnea (CSA) is sleep disordered breathing in which there is an absence or diminution of respiratory effort during breathing disturbances while asleep. In obstructive sleep apnea (OSA), on the other hand, there is an absence of flow despite ongoing ventilatory effort.

**Recent Findings:**

Central sleep apnea is a heterogeneous disease with multiple clinical manifestations.

**Summary:**

OSA is by far the more common condition; however, CSA is highly prevalent among certain patient groups. Complex sleep apnea (CompSA) is defined as the occurrence/emergence of CSA upon treatment of OSA. Similarly, there is considerable overlap between CSA and OSA in pathogenesis as well as impacts. Thus, understanding sleep disordered breathing is important for many practicing clinicians.

## Introduction

**Fig. 1:**
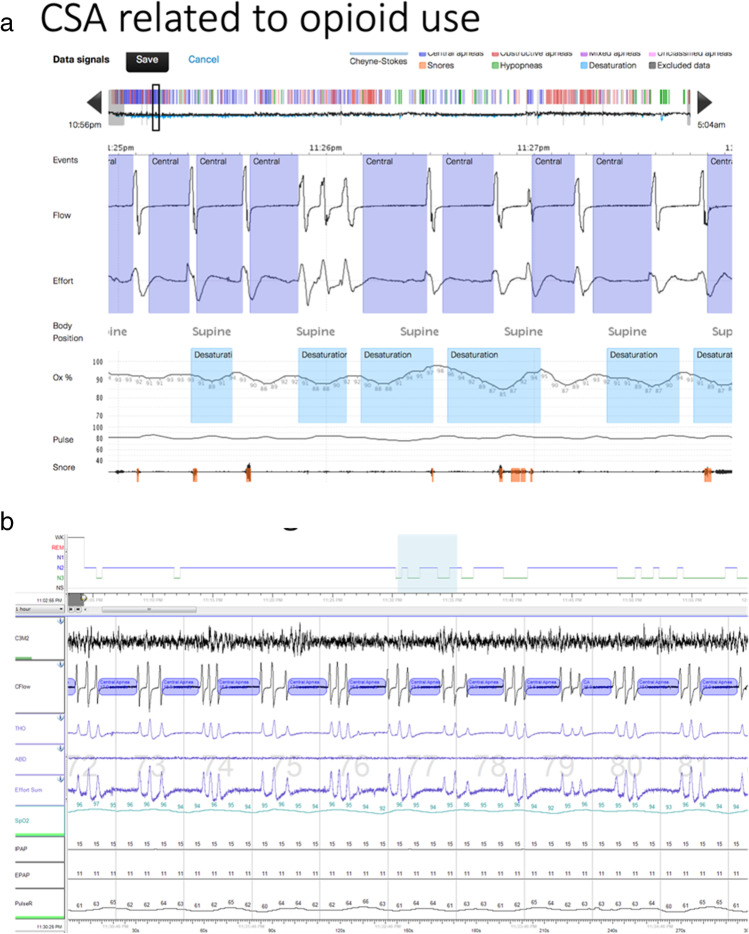
Polysomnography in central sleep apnea. **a** The above tracing shows central sleep apnea with ataxic breathing pattern. Note the absence of airflow without respiratory effort characteristic of central apnea. The oximetry shows associated desaturations. **b** The tracing shows the EEG (C3M2), Cflow (airflow), thoracic (THO), and abdominal (ABD) belts to assess respiratory effort and the IPAP (inspiratory positive airway pressure) and EPAP (expiratory positive airway pressure) being provided via machine. Note again the absence of airflow without respiratory effort defining central apnea

OSA is estimated to affect up to 1 billion people worldwide [1]. The epidemiology of CSA is less well studied, but the prevalence of CSA is estimated to be 5 to 10% of patients with SDB [2, 3]. Central sleep apnea has a number of etiologies that also have varying underlying mechanisms, and broadly can be attributed to either inadequate ventilatory drive or a paradoxically excessive drive (elevated loop gain) [4, 5, 6, 7, 8]. One of the most recognized forms of CSA is Cheyne-Strokes breathing in patients with heart failure who have high ventilatory drive [9, 10]. Central sleep apnea is also seen in patients at high altitude, with certain medications (such as opioids), or is uncovered when OSA is treated (complex sleep apnea, CompSA) [11]. CSA has profound clinical implications as it leads to arterial oxygen desaturation, hypercapnia, arousals from sleep, surges in ventilatory drive, and sympathetic excitation [12]. In adults, CSA is often defined as the presence of at least 5 central events per hour, which can include central apneas or central hypopneas. For patients with heart failure, criteria vary but the diagnosis of CSA is typically at least 15 events per hour with at least 50% of events being central [13]. Example polysomnography (PSG) tracings are found in Fig. 1. However, since effort is not directly measured (such as via diaphragm EMG or esophageal pressure), identifying central events (particularly hypopneas) can be difficult, leading to CSAs being underreported.

## Pathogenesis of CSA

Ventilatory control is regulated by a feedback loop involving chemoreceptors. At the carotid bodies in the bifurcation of the internal and external carotid arteries, increased PCO2 and reduced PO2 are sensed quickly due to abundant perfusion to the region, comprising the peripheral chemoreceptors. Meanwhile, the medulla and pons serve as the central chemoreceptors by sensing increased PCO2 in the form of H^+^. The central chemoreceptors determine the baseline ventilatory effort, and both peripheral and central chemoreceptors provide feedback to generate the strength and frequency of motor output to the diaphragm and external intercostal muscles. Controversy remains regarding the interactions between central and peripheral chemoreceptors: Dempsey et al. have proposed a hyperadditive model whereby the receptors can amplify the gain on one another during various perturbations [14, 15, 16].

The concept of loop gain is being increasingly used in the context of control of breathing [17, 18]. Loop gain is an engineering term which describes the stability or instability of a feedback control system. A system with a high loop gain is one that is prone to instability whereas a system with low loop gain is one that is intrinsically stable. The regulation of carbon dioxide in the body is a negative feedback control system that is attempting to keep the PaCO2 at roughly 40 mmHg. Major fluctuations in PaCO2 reflect a high gain on the system. The analogy to room temperature can be helpful whereby various factors can be important in maintaining a constant room temperature. For a given room with a temperature at 20 °C, the temperature in the room would oscillate if the thermostat were too sensitive. That is for a minimal increase in temperature, a major increase in air conditioning could lead to marked fluctuations in room temperature, thus, an unstable control system with fluctuating room temperatures. By analogy, an overly sensitive carotid body could lead to marked changes in ventilation for trivial changes in PaCO2 and thus an unstable control system with major fluctuations in PaCO2[19]. Another situation that could lead to marked fluctuations in room temperature would be a furnace that was too powerful. For example, a fall in room temperature to 19 °C might make an overly powerful furnace lead to an increase in room temperature to 60 °C which would be a situation with markedly unstable room temperature and thus an unstable control system [20]. By analogy, the PaCO2 could become unstable if a minor increase in PaCO2 to 41 mmHg led to an overly exuberant response dropping the PaCO2 to 10 mmHg. This over-responsiveness could lead to marked fluctuations in PaCO2 and thus an unstable control system. The controller gain is defined by the component of the overall loop gain that is related to chemoresponsiveness, i.e., in part mediated by chemosensitivity. The plant gain is another component of the overall loop gain which is defined by the efficiency of CO2 excretion. The products of the controller gain and plant gain are generally what constitutes the overall loop gain [21, 22, 23].

The prototype for high loop gain is the clinical scenario of Cheyne-Stokes breathing (CSB) whereby individuals, commonly with congestive heart failure, have fluctuations in breathing as a function of CO2 fluctuations [24, 25, 26]. The CSB patients have high loop gain as a result of robust chemosensitivity [19] and other factors which can be addressed clinically. Importantly, it should be recognized that CSA related to high loop gain is due to a hyperresponsive system associated with high respiratory drive, and not respiratory control failure leading to low drive.

Some controversy exists regarding the importance of circulatory delay towards unstable breathing [4, 18, 27, 28]. The absence of important chemoreceptors in the lung allows the situation whereby changes in ventilation can occur that are not perceived by chemoreceptors until overshoots and undershoots have occurred. Indeed, Guyton et al. [29] induced circulatory delay in some animal models and were able to induce periodic breathing with prolonged circulatory delays. However, the delays in some cases were several minutes long, i.e., outside the range that would be considered clinically relevant [30]. In studies that have compared circulatory delay in heart failure patients with and without CSB, in general circulatory delay is similar in the 2 groups suggesting that the delay is not a critical factor in most cases [12]. On the other hand, heart failure is associated with longer delays [31], and improvement in circulatory delay, e.g., via cardiac resynchronization therapy (CRT), leads to small improvements in apnea. Thus, in aggregate, the data suggest that some amount of circulatory delay is necessary, but delays alone are not sufficient to induce unstable breathing patterns in most clinical situations [27].

In addition to CSB, some patients exhibit central apneas with fluctuating alertness, i.e., so-called state transition apneas. In this context, patients who transition from wake to sleep back to wake may exhibit central apneas as a result of so-called state instability. In this setting, a ventilatory response to arousal can occur where an individual who was asleep with a PaCO2 of 45 mmHg might drive the CO2 to low levels upon awakening based on the ventilatory response to arousal [32, 33, 34, 35, 36, 37]. For example, CSA occurs more commonly at sleep onset or in stage 1 non-REM sleep compared with stable wakefulness or deeper N3 sleep. As a result, sedatives can actually improve CSA in some patients if the medication limits state instability.

In some patients with hypercapnia, central apneas/hypopneas can occur during sleep. Three clinical scenarios include hypercapnic chronic obstructive pulmonary disease, obesity hypoventilation syndrome, and neuromuscular disease [38, 39]. In these patients, the loss of the wakefulness drive to breathe at sleep onset can lead to worsening hypoventilation [32, 40]. In such scenarios central apneas and hypopneas can frequently occur. In rare congenital forms, CCHS (central congenital hypoventilation syndrome) can occur as a result of a mutation in the phox2b gene. Such patients can present at birth with cyanosis although more subtle forms have been diagnosed in adulthood [41, 42]. CSA in this scenario is associated with low respiratory drive (or severe neuromuscular weakness) rather than excessive drive along with high loop gain.

## CSA Syndromes

Central sleep apnea includes 6 individual syndromes according to the International Classification of Sleep Disorders 3rd edition (Table 1).

**Table 1:** CSA syndromes

Type	Cause	Treatment
Cheyne-Stokes breathing	Elevated loop gain	Optimize heart failure medications
Complex sleep apnea	Relief of upper airway obstruction	Expectant; occasionally ASV if needed
Primary CSA	Idiopathic	Consider ASV or bi-level with backup rate
High altitude periodic breathing	Alveolar hypoxia; high gain	Oxygen, acclimatization
CSA from medication	Variable, commonly opioid induced high gain	Reduce medications, supportive care, consider ASV
CSA from comorbidity (non-CSB)	Variable	Treat underlying cause

### Cheyne-Stokes Breathing (CSB)

Cheyne-Stokes breathing occurs in a large proportion of patients with heart failure and is a well-described phenomenon [43]. It is a waxing and waning pattern of breathing with frequent periods of central apnea. It is present in 20–40% of patients with left ventricular systolic dysfunction and is a common clinical manifestation of central apnea. For example, patients with worsened systolic function, reduced cardiac output, and atrial fibrillation are more likely to have CSA [44], whereas cardiac resynchronization and afterload reduction improve ventilatory stability [45, 46]. However, as previously noted, delayed circulation alone is not sufficient to explain this occurrence and, rather, the combination of increased chemosensitivity in conjunction with delayed circulation is much more likely to result in CSA. The etiology of increased chemosensitivity is not well known and may have multiple causes such as elevated pulmonary capillary wedge pressures, overnight fluid shifts from the extremities to the pulmonary vasculature, or abnormalities of the carotid bodies themselves [47, 48, 49]. Intermittent hypoxemia and catecholamine surges lead to neuroendocrine activation and oxidative stress, which likely worsens the underlying heart failure. Cheyne-Stokes breathing may manifest in the form of paroxysmal nocturnal dyspnea for many patients as they tend to awaken during hyperpneas [50, 51].

Management of CSA with Cheyne-Stokes breathing should include optimization of treatment for the underlying heart failure. Continuous positive airway pressure (CPAP) can improve breathing indices, but has not definitively been shown to improve mortality [52]. Nonetheless, based on post hoc analysis suggesting improved outcomes in those who respond to CPAP (i.e., normalization of the AHI) [53], as well as mechanistic data suggesting improved heart failure physiology, CPAP can be used with the goal of normalization of the AHI [54]. Adaptive servoventilation (ASV) is a form of non-invasive ventilation that is highly efficacious at reducing the AHI by improving ventilatory instability. Nonetheless, a large randomized clinical trial demonstrated adverse outcomes with use of ASV in heart failure with reduced ejection fraction, prompting a black box warning of ASV in CHF with CSB and reduced ejection fraction [55]. Ongoing studies are further examining the potential role of ASV in heart failure including those with preserved ejection fraction [56, 57, 58, 59, 60, 61]. Supplemental oxygen can suppress CSB for some patients, although definitive data are lacking, and narrow insurance coverage criteria can limit the ability to obtain this therapy. Other strategies under investigation that have demonstrated some improvements in AHI and other parameters include pharmacotherapy (e.g., acetazolamide [58, 62•, 63, 64, 65] and buspirone) as well as phrenic nerve stimulation [66], although hard outcome data are lacking.

### Complex Sleep Apnea (CompSA)

This phenomenon (sometimes called “treatment emergent CSA”) has been observed once the upper airway has been made patent with treatment, such as CPAP therapy or tracheostomy. Approximately 10% of patients with obstructive sleep apnea (OSA) also clinically demonstrate CSA during CPAP titration studies, which can contribute to poor adherence with therapy [67]. For patients adherent with CPAP therapy, residual apnea can remain in up to 4% of patients and its optimal treatment remains unclear. A potential mechanism is the relief of inspiratory flow limitation allowing the unmasking of a high chemosensitivity leading to central sleep apnea [68•]. In terms of treatment, switching from CPAP to adaptive servo ventilation (ASV) is a potential solution and is associated with improvements in apnea [69] and possibly adherence with therapy [68•]. Furthermore, ASV therapy had excellent adherence as well as associated with lower AHI. However, randomized trials will be required to draw definitive conclusions.

### Primary CSA

Primary or idiopathic CSA occurs without any identifiable cardiac or neurological cause or medication use that could induce CSA. Similar to other CSA syndromes, polysomnography (PSG) shows 5 or more respiratory events per hour of sleep and the number of central apneas or central hypopneas > 50% of the total. In contrast to CSB, the cycling period is shorter, generally lasting between 30 and 40 s, likely reflecting the lack of substantial circulatory delays in primary CSA [70]. Increased chemosensitivity and ventilatory overshoot likely play a role in primary CSA, and sleep tends to be quite fragmented. Treatment is not well established, but many clinicians will offer CPAP, ASV, or other non-invasive ventilation. Note that bilevel ventilation (“BIPAP”) without a backup rate has the potential to worsen central apneas.

### High-Altitude Periodic Breathing

Decreased sleep quality and mood as well as impairments in cognitive function occur after ascent to high altitude [71]. Central sleep apnea occurs in practically all people at arrival to high altitude (e.g., elevations above 2500 m). The stimulus to CSA in this context is low total barometric pressure with a stable fraction of oxygen leading to a decreased inspired partial pressure of oxygen. The hypoxia leads to augmentation of resting ventilation as well as the chemoreflex response to CO2. Moreover, over time ventilatory acclimatization leads to further increases in the chemoreflex response, although whether CSA persists or resolves may depend on the altitude as well as factors such as genetic background. The ventilatory cycle of high altitude periodic breathing is usually between 12 and 34 s and is characterized by alternating hyperpnea and apnea during non-rapid eye movement (NREM) sleep [70]. Additionally, central sleep apnea is associated with hypoxemia and pulmonary hypertension that may yield chronic mountain sickness.

A common treatment for acute mountain sickness and CSA at altitude is nighttime supplemental oxygen, which decreases the apnea–hypopnea index (AHI) and improves sleep quality. Another potential treatment is adaptive servoventilation (ASV), which is not as effective as supplemental oxygen at improving nighttime oxygen saturations, but can improve sleep quality [72]. One study compared nighttime supplemental oxygen to ASV to placebo at high altitude and had subjects complete a cognitive test battery as well as mood and sleep quality questionnaires the following morning. Both nighttime supplemental oxygen and ASV improved levels of daytime fatigue and confusion at high altitudes, but did not improve any other measures of cognitive performance [70]. ASV did not improve sleep quality, but supplemental oxygen decreased periodic breathing and arousals as well as improved mean nighttime saturation. However, there is a concern that supplemental oxygen may slow the acclimatization process.

Acetazolamide is another potential treatment for CSA due to altitude. In a meta-analysis of short-term acetazolamide for treatment of both OSA and CSA, there was a reduction in AHI as well as an improvement in SpO2 nadir [62•]. It was effective for both OSA and CSA, but the biggest benefits were seen in studies of CSA at high altitudes and at higher doses of acetazolamide up to 500 mg/day. Acetazolamide treatment was also associated with increased sleep duration, decreased arousal index, and a shift towards deeper sleep stages. However, acetazolamide has also been associated with impaired memory and reduced processing speed and concentration following ascent compared to controls [73]. More studies of acetazolamide, especially long-term use, are warranted to investigate further.

### Central Sleep Apnea due to a Medication or Substance

Opioid-induced CSA is a relatively newly recognized phenomenon and studies suggest that approximately one-third of patients on chronic opioids have some degree of CSA [74•]. Opioids are associated with bradypnea, hypoventilation, hypercapnia, and hypoxemia as well as erratic breathing patterns. The cycle period of CSA from opioids is very similar to high-altitude; studies suggest that similar elevations in loop gain are present in these two conditions. Possible etiologies of the elevated loop gain include reduced CO2 damping from an elevated alveolar PCO2, increased hypoxic chemosensitivity due to severe hypoventilation and concomitant hypoxemia, and a doubling of the slope of the hypoxic ventilatory response independent of hypoxemia [75]. Elevations in venous CO2 are thought to increase the efficiency of CO2 excretion thus leading to elevated plant gain. Treatment can be challenging for opioid-induced CSA. CPAP is not consistently effective for treatment, but it may be possible that reducing opioid doses may normalize the breathing pattern [2]. ASV, similar to treatment emergent CSA, has been effective in small studies [76].

### CSA due to a Medical Condition Without Cheyne-Stokes Breathing

This pattern usually occurs in patients with cardiac, renal, or neuromuscular disease, but who do not demonstrate Cheyne-Stokes breathing. Examples include chronic obstructive pulmonary disease, interstitial lung disease, idiopathic pulmonary hypertension, and chronic thromboembolic disease with pulmonary hypertension. The etiology of CSA depends on the underlying condition and may include physiology driving high loop gain (e.g., fluid overload related to renal failure), or alternatively conditions associated with low respiratory drive and/or weakness (e.g., chronic lung disease, diaphragm paralysis) [77].

In the context of neurological diseases, there is no causal pathway between sleep apnea and neurodegeneration, but there are a number of findings and associations that deserve mention. First, some associations have been reported between Parkinson’s disease and central apnea although the mechanisms are not clearly delineated. Other causes of neurodegenerative disease (including Alzheimer’s) have been anecdotally associated with central sleep apnea although the majority of the data suggest that obstructive sleep apnea is far more common in this context [78]. At least in theory, neurodegeneration effects on the central pattern generation (i.e., pre-Botzinger complex) in the brainstem could contribute to central apnea, although further work is clearly needed in this area [79]. Second, in patients with fluctuating levels of consciousness, variations in PaCO2 can lead to central apneas. Similar to the state transition apneas mentioned above, the wakefulness drive to breathe can contribute to a fall in CO2 below the chemical apnea threshold during these state transitions [32]. Thus, neurological conditions may contribute to central apnea through so-called state instability [2]. Third, in the context of neuromuscular diseases (such as amyotrophic lateral sclerosis), hypoventilation commonly occurs particularly during REM sleep when skeletal muscle atonia occurs in accessory muscles of respiration. The resulting loss in muscle tone can contribute to hypopneas as a result of reduced minute ventilation due to reduced neuromuscular activity in respiratory muscles [80, 81]. Fourth, common risk factors are frequently present for central apneas and neurological diseases. For example, patients with atrial fibrillation and congestive heart failure can have elevated loop gain and thus risk of central apnea but such individuals are at also at risk of cardioembolic stroke [82, 83, 84]. In theory, the co-occurrence of various comorbidities (e.g., congestive heart failure with Parkinson’s disease) might contribute synergistically to risk of central apnea. Ultimately, although central respiratory events during sleep are seen in neurological diseases, the majority of central apnea observed clinically is from other etiologies.

## Conclusions

Central sleep apnea is a heterogeneous and complex disease characterized by the temporary absence of ventilatory effort during sleep. In the majority of cases, it is a result of conditions leading to an elevated loop gain, sometimes driven by a robust ventilatory effort in response to perturbation. Given the variety of etiologies and mechanisms leading to similar clinical syndromes, response to available treatments has often been mixed. However, there are emerging novel treatment strategies including adaptive/auto servo ventilation, phrenic nerve stimulation, and pharmacotherapy. Further studies to understand the pathophysiology more rigorously could eventually yield improved treatments and have major impact on patient care.
